# Cutaneous Leishmaniasis in North Africa: a review

**DOI:** 10.1051/parasite/2014014

**Published:** 2014-03-14

**Authors:** Karim Aoun, Aïda Bouratbine

**Affiliations:** 1 Institut Pasteur de Tunis, Laboratoire de Parasitologie, 13 Pl Pasteur BP 74 Tunis 1002 Tunisia

**Keywords:** *Leishmania infantum*, *Leishmania major*, *Leishmania tropica*, Epidemiology, Algeria, Libya, Morocco, Tunisia, Egypt

## Abstract

In North African countries, cutaneous leishmaniasis transmission has been increasing since the 1980s, with a significant increase in the incidence of cases and a spread of the geographical distribution. The disease currently represents a major public health problem with a productivity gap and an impediment for development, which results in dramatic socioeconomic and psycho-sanitary impacts. The incidence is more than thousands of cases every year in Algeria, Libya, Morocco, and Tunisia. In Egypt, only a few dozen cases per year are reported, mainly in the Sinai Peninsula. Three *Leishmania* species, associated with distinct eco-epidemiological and clinical patterns, are involved, namely *Leishmania infantum*, *L. major*, and *L. tropica*. However, *L. major* is by far the most frequent in Algeria, Libya, and Tunisia, with more than 90% of the registered cases. It is mainly encountered in rural areas under semi-arid, arid and Saharan climates. *Leishmania tropica* is more prevalent in Morocco, reaching 30–40% of isolates in some districts. Much data is still missing concerning the risk factors of the infection and the lesion development, as well as vector and reservoir ecology and behavior. The knowledge of such parameters, following multidisciplinary and integrated approaches, is crucial for better management and control of the disease, that also faces a lack of resources and efficient control measures.

## Abstract (fr)

La leishmaniose cutanée est émergente en Afrique du Nord depuis le début des années 80 avec une augmentation importante de l’incidence des cas et une extension de la répartition géographique. La maladie constitue un problème majeur de santé publique, avec un impact négatif sur la productivité et un handicap pour le développement, qui altère les conditions socio-économiques et psycho-sanitaires. L’incidence dépasse les milliers de cas chaque année en Algérie, Libye, Maroc et Tunisie. Elle est plus faible en Égypte, limitée à quelques dizaines de cas principalement dans le Sinaï. Trois espèces de *Leishmania*, associées à des caractéristiques environnementales, épidémiologiques et cliniques distinctes, sont responsables de la maladie, à savoir *Leishmania infantum*, *L. major* et *L. tropica*. *Leishmania major* est de loin la plus fréquente en Algérie, Libye et Tunisie avec plus de 90 % des cas enregistrés. Elle sévit principalement dans les zones rurales des étages bioclimatiques semi-arides, arides et sahariens. *Leishmania tropica* est plus répandue au Maroc où elle est responsable de 30 à 40 % des cas dans certaines régions. De nombreuses données manquent encore concernant les facteurs de risque de l’infection, la pathogénie des espèces en cause, et l’écologie et le comportement des vecteurs et réservoirs. La connaissance de ces paramètres, grâce à une approche multidisciplinaire et intégrée, est fondamentale pour un contrôle de la maladie, qui reste par ailleurs également confronté aux insuffisances des ressources et des mesures efficaces de lutte.

## Abstract (ar)

داء اللايشمانيا الجلدي من الأمراض الناشئة في بلدان شمال إفريقيا والعدد الإجمالي للحالات في ارتفاع متواصل مع انتشار جغرافي للوباء منذ بداية الثمانيات. يعد هذا الداء من المشاكل الصحية ذات الأولوية في المغرب العربي ولو انه اقل حدة في القطر المصري ولهذا الداء تأثيرات مباشرة صحية واجتماعية واقتصادية، كما له انعكاسات سلبية على النمو والتطور في البلدان المتضررة. هناك ثلاثة أنواع من طفيلي اللايشمانيا تتسبب في المرض في شمال إفريقيا و ترتبط كل منها بخاصيات بيئية و وبائية و سريرية، وهي اللايشمانيا الطفيلية ( *L. infantum *) و اللايشمانيا الرائدة ( *L. major *) و اللايشمانيا المدارية.( *L. tropica *) اللايشمانيا الرائدة هي الأكثر انتشارا بتونس والجزائر وليبيا حيث تتسبب في أكثر من %90 من الحالات المرضية و تتوزع هده الحالات بالأخص في المناطق الريفية و القاحلة والصحراوية. و تختص الوضعية الوبائية في المغرب بارتفاع نسبي لعدد الحالات المنسوبة لللايشمانيا المدارية (30 إلى %40) في حين تبقى اللايشمانيا الرائدة أكثر تفشيا. البحوث العلمية والحملات الميدانية يجب أن تكثف لتحسين وإتمام المعلومات خاصة تلك المتصلة بالخازن و الفواصد الناقلة وخصوصياتها البيئية. لهذه المعلومات أهمية قصوى في مجابهة هذا المرض الذي يبقى التغلب عليه رهن توفير الإمكانيات المادية واللوجستية من ناحية والحلول الفعالة من ناحية أخرى.

## Introduction

Cutaneous leishmaniasis (CL) is a parasitic vector-borne disease caused by the flagellate protozoa of the genus *Leishmania* and transmitted by the bite of female sandflies. It is caused by a variety of *Leishmania* species, each one having specific mammal reservoir hosts and vectors [56]. In humans, infection results in a cutaneous lesion at the site of the infective sandfly bite. The clinical characteristics of CL vary according to the infecting *Leishmania* species. However, a single species can even produce lesions with different characteristics in the same person [53]. Cutaneous leishmaniasis persists for months, or even years in some cases, before the lesions heal spontaneously and leave flat, hypopigmented and atrophic scars [53]. Although CL is usually self-curing and not life-threatening, infected patients may be psychologically and socially damaged. In fact, it is not unusual for several lesions to cause great disfigurement and distress.

Cutaneous leishmaniasis is endemic in many regions of the world such as Latin America, the Mediterranean basin, and western Asia from the Middle East to central Asia [2]. In North Africa (NA), the disease is highly prevalent in Morocco, Algeria, Tunisia, and Libya, where the emergence of new endemic foci with a consequent drastic increase in CL incidence have been reported since the 1980s. The number of cases remains low in Egypt [2]. In NA countries, CL cases are caused by three *Leishmania* species: *Leishmania (Leishmania) major, Leishmania (Leishmania) tropica*, and *Leishmania (Leishmania) infantum.* These three species prevail under different bioclimates and differ by the nature of their vectors and reservoir hosts. They are responsible for three nosogeographical CL forms which show different epidemiological and clinical features and need specific control measures.

Here, we summarize current knowledge on CL prevalence, transmission cycles, major clinical manifestations, and therapeutic approaches in NA countries. Cutaneous leishmaniasis incidence rates and control strategies are also described, focusing on particular aspects in each NA country. Epidemiological and clinical information were assessed by a review of the literature using the online database PubMed with medical subject headings, cutaneous leishmaniasis, and each of the North African countries. This information was completed by institutional (Ministries of Health records) data. Several local studies were recorded. A number of these studies offer extended and reliable information about some countries, being nationwide or using large and representative samples. However, for some countries available and reliable data may be lacking.

## Epidemiology and transmission cycles of causative *Leishmania* species

### Leishmania major

**Figure 1.**
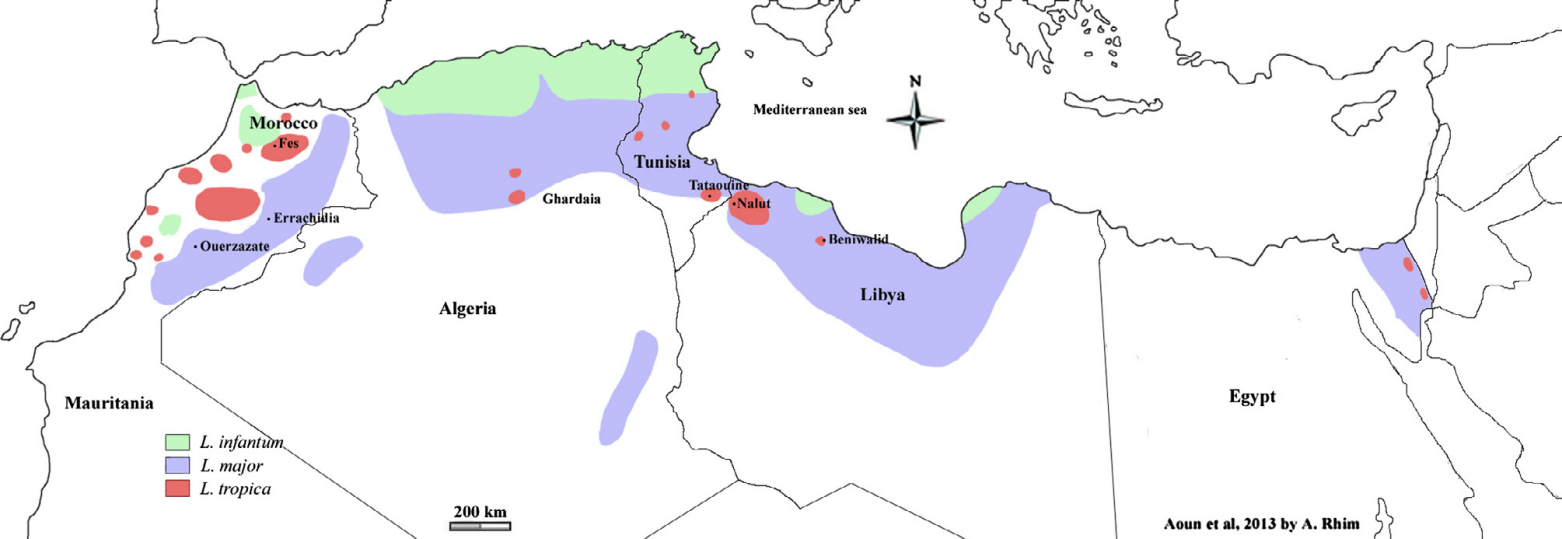
Geographical distribution of cutaneous leishmaniasis cases due to *L. infantum*, *L. major*, and *L. tropica* in North African countries.

*Leishmania major* has a high incidence rate in all NA countries where it is considered as a public health threat. Zoonotic CL (ZCL) is distributed in the arid and Saharan bioclimatic stages where it follows an epidemic pattern with seasonal occurrence of cases (Fig. 1). Zoonotic CL incidence and the interval between epidemics depend on climatic factors [67].

The transmission life cycle of *L. major* in NA is well established. *Leishmania major* MON-25 is the exclusive zymodeme responsible for the disease [5, 48, 54]. Only a single Algerian isolate, identified as *L. major* MON-269, does not correspond to this zymodeme [54]. *Phlebotomus (P.) papatasi* is the proven vector. It frequently uses rodents’ burrows for daytime resting and breeding [17, 39, 45]. The fat sand-rat *Psammomys (P.) obesus* and the gerbil *Meriones* spp. are reservoir hosts [10, 11] (Table 1).

**Table 1.** Vectors and reservoir hosts of *Leishmania* species involved in cutaneous leishmaniasis in North African countries.

*Leishmania* species	Vector	Reservoir host
*L. major* Yakimoff and Schokhor, 1914	*Phlebotomus papatasi*	*Psammomys obesus, Meriones shawi, M. libycus*, Egypt: *M. crassus*
*L. tropica* Wright, 1903	*P. sergenti*	Morocco: Man, Domestic dog? Egypt: *Gerbillus pyramidum*?
*L. tropica* MON-8 (*L. killicki* Rioux, Lanotte & Pratlong, 1986)	*P. sergenti*	Tunisia: *Ctenodactylus gundi* Algeria, Libya: Probably gundi
*L. infantum* Nicolle, 1908	*P. perfiliewi, P. perniciosus, P. longicuspis, P. ariasi*	Domestic dog

*Psammomys obesus* is distributed through the semi-desert on the northern fringe of the Sahara, from Mauritania through Morocco, Algeria, Tunisia, Libya, and Egypt to the Middle East. It lives in densely packed burrows, in saline habitats, notably “succulent halophytic steppes”, or along wadi edges, where its main food, plants of the family Chenopodiaceae, grows. These chenopods constitute its strict diet and govern its local distribution and abundance. In fact, high sand-rat densities are associated with abundant vegetation [10, 11]. *Psammomys obesus* is the main reservoir host of *L. major* [10]. It was demonstrated that it was naturally infected in Libya [12], in Algeria [14, 20], and in Tunisia [15, 33, 34].

The gerbils, *Meriones* spp., may play a role at least temporarily in the maintenance of the disease [10]. They also may have a role in the propagation of the parasite to populated areas where a population surge of *Meriones* is associated with CL outbreaks in humans [61]. *Meriones* jirds tend to inhabit arid regions including clay or sandy desert and steppes, but are also found in slightly wetter regions. This rodent is considered as an agricultural pest. *Meriones (M.) shawi* is the main infected animal in south Morocco [61]. It was also found to be naturally infected in both Algeria [20] and Tunisia [34, 62]. *Meriones libycus* was also found to be infected by *L. major* in both Tunisia and Libya [12, 16] and *M. crassus* in Egypt [52] (Table 1).

Zoonotic CL was historically largely confined to several oases located in arid pre-Saharan regions of NA, where the disease was typically sporadic and occasionally followed an epidemic pattern. Since the 1980s, a reactivation of old foci along with an expansion of ZCL beyond its natural eco-region have been observed in Libya, Algeria, Tunisia, and Morocco. Consequently, outbreaks amounting to several thousand cases have been repeatedly reported. The start of these large epidemics can be explained by climatic change. In fact, some authors reported shifts in the surface climate that may have changed the dynamic of ZCL in the pre-Saharan NA zones [21]. In addition, epidemiological modifications can be explained by the ecological and environmental changes that have happened in the NA region these last decades. In fact, in the Saharan region, the replacement of the camel (the only competitor with *P. obesus* for the halophilic plants on which it depends) with motor vehicles in many relevant areas may be responsible for the vegetation flourishing, the increase in sand-rat numbers, and the extension of the range of both the rodent and disease [11]. In arid areas, the huge mobilization of water resources and the creation of new irrigated zones probably favored the proliferation of both sandflies and rodents, including gerbils and jirds living in burrows with *P. papatasi*, the vector of *L. major* [4]. On the other hand, ZCL cases have probably emerged in new areas as a result of establishment of high densities of susceptible human populations near to *L. major* reservoir host biotopes, such as salt marshes invaded by chenopods, jujube, and rodent burrows. Clustering of infections within these settlements suggests a spread and an adaptation of transmission cycles to a peridomestic environment [24]. In fact, owing to its specific behavior, *P. papatasi* is able to adapt well to anthropogenic settings [24, 66].

### Leishmania tropica

*Leishmania tropica* presents its largest geographic distribution and highest incidence rate in Morocco. It is found throughout the center of the country in a band stretching from the Atlantic Ocean along the length of the Atlas Mountains almost to the Mediterranean Sea [57] (Fig. 1). The North African *L. tropica* CL form remains clearly distinguishable from the anthroponotic urban and hyper-endemic CL observed in cities of the Middle East and Central Asia, namely Aleppo-Syria or Kabul-Afghanistan [56]. In fact, this CL form prevails in scattered hypo-endemic rural foci and small towns located in the arid mountains where the disease is typically sporadic. However, in recent years, several epidemics with hundreds of cases have been reported in several Moroccan cities [57].

*Leishmania tropica* is recognized as a very heterogeneous species and its intraspecific heterogeneity is readily demonstrated by many authors. However, its genetic and isoenzyme diversity varies according to the NA country concerned. In fact, in terms of isoenzyme profile variability, Morocco is characterized by the largest number of zymodemes ever described in *L. tropica*, with eight zymodemes (*L. tropica* MON-102, MON-107, MON-109, MON-112, MON-113, MON-122, MON-123, and MON-279) detected from human CL, dogs, and sandflies [29, 50, 55]. However, in Algeria, Tunisia, and Libya less diversity is observed, with the presence of only *L. tropica* MON-8 (syn *L. killicki*) in both Tunisia [5] and Libya [9] and *L. tropica* MON-301 (which is also related to *L. killicki*) in Algeria [42]. In 1986, *L. killicki* was described as a new species [60]. However, currently all *L. tropica* zymodemes, the *L. killicki* zymodeme (MON-8), and the zymodeme related to *L. killicki* (MON-301) are considered as belonging to a single broad *L. tropica* complex [54]. On the other hand, microsatellite analysis revealed that two genetically very distinct populations of *L. tropica* co-exist within the same focus in Morocco: population “Morocco A” is related to population “Asia”, whereas population “Morocco B” is genetically closer to the other African populations. This data suggests both anthroponotic and zoonotic transmission cycles of the parasites present in the same Moroccan CL foci [63].

Much debate concerns the transmission cycle of *L. tropica* in NA (Table 1). In Morocco, the disease is often described as being anthroponotic [37, 57]. However, the epidemiology of *L. tropica* in this country is by no means fully understood. In particular, sporadic cases occur in rural areas and small towns, which cannot readily be explained by local anthroponotic transmission. Either these latter are dependent foci in which the parasite requires repeated introduction, or there may be unidentified zoonotic sources. [11]. This idea is supported by the greater diversity of *L. tropica* strains in the proven vector *P. sergenti* compared with the diversity observed in humans [1, 38]. Thus, the precise role of humans, domestic dogs [36], and other animals as reservoir hosts is not well established. In Algeria, Tunisia, and Libya, the relative paucity of CL cases and their spatial distribution excludes the anthroponotic character of the disease [24, 42]. *Phlebotomus sergenti* is the proven vector [19, 46, 65]. However, other sandfly species may contribute to the transmission cycle [66]. The putative animal reservoir is the gundi [9, 25, 42]. *Ctenodactylus gundi* is extremely abundant in all Tunisian *L. tropica* foci, where it is found in natural and peridomestic environments. Moreover, it was found to be infected by *Leishmania* using PCR [25, 47]. In addition, molecular analysis indicated that similar genotypes were present in humans, in *P. Sergenti* naturally infected by *L. tropica* promastigotes, and gundis from the same region [25].

### Leishmania infantum

Besides the dermotropic species *L. major* and *L. tropica*, the viscerotropic species *L. infantum* is also involved in CL in NA. The CL form due to *L. infantum* is distributed in the Northern part of the different countries (Fig. 1) in humid, sub-humid, and semi-arid bioclimatic stages. It prevails as sporadic cases in the area of distribution of visceral leishmaniasis.

Isoenzyme analysis of *L. infantum* NA strains shows that zymodeme MON-24 is the most frequently isolated from cutaneous lesions, whereas the viscerotropic zymodeme MON-1 and the zymodeme MON-80 are less commonly identified in CL cases [5, 8, 43, 48]. Phlebotomine species vectors belong to the subgenus *Larroussious*, namely *P. perfiliewi*, *P. perniciosus*, *P. longicuspis*, and *P. ariasi*, and vary according to the countries and areas concerned [35, 43]. The domestic dog is the putative reservoir host. However, *L. infantum* MON-24 is still rarely isolated from dogs [18] (Table 1).

## Clinical features and treatment approaches

The typical CL lesion first appears as an erythematous papule at the site where promastigotes are inoculated, slowly increases in size, becomes a nodule, and eventually ulcerates [53]. In endemic regions, chronic ulcerative lesions with scabs are highly suggestive of CL. However, many other clinical pictures can be observed.

According to species, the necrotic process may be rapid, causing a large, open, wet sore or may be more indolent, without frank ulceration. Similarly, the size and location of the lesions may vary. Spontaneously healing without treatment is also the rule, but the time needed greatly varies according to parasite identity.

**Figure 2.**
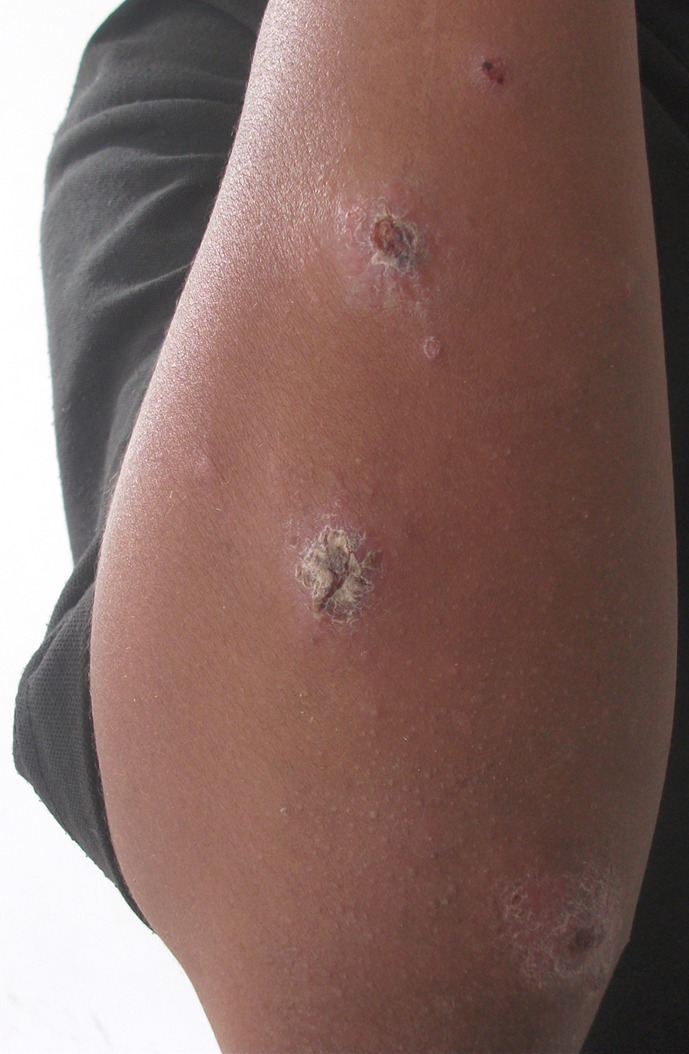
Multiple ulcerous lesions with scabs of the forearm due to *L. major*.

*Leishmania major* causes cutaneous lesions that tend to be exudative or “wet”, large and complicated by superficial and secondary bacterial infections. Zoonotic CL lesions are typically multiple and located on limbs (Fig. 2). Peaks of emerging cases are observed in fall, mainly between September and November [6]. Spontaneous healing but with indelible scars is obtained in less than 8 months [6, 24, 26].

**Figure 3.**
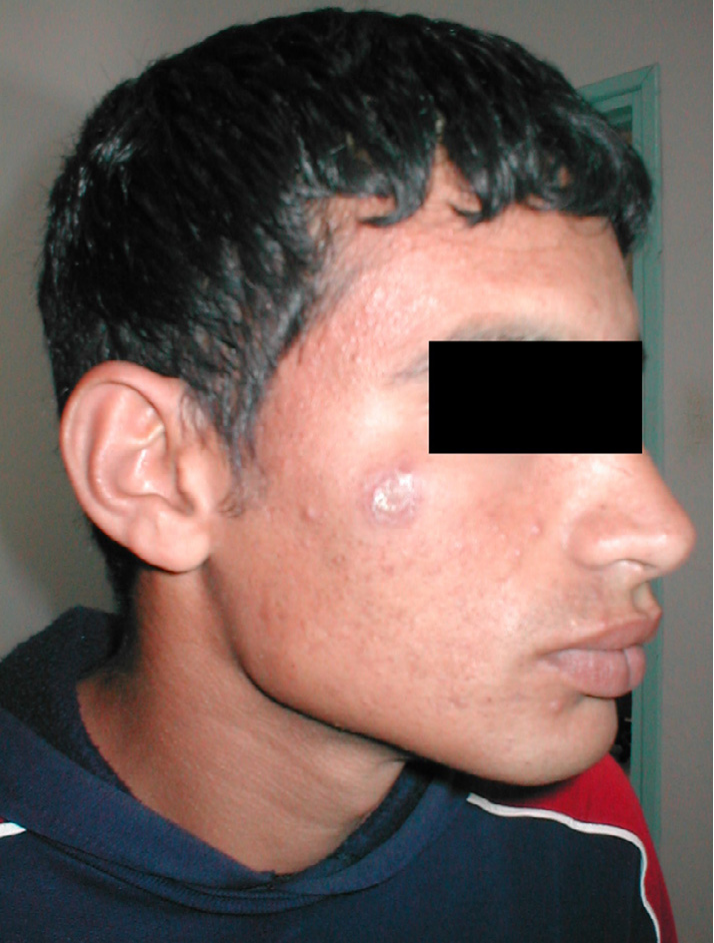
Dry and slightly inflammatory single lesion of the face due to *L. tropica*.

Lesions caused by *L. tropica* are often “dry” with a central crust. They are mainly single and located on the face (Fig. 3). Some lesions last more than one year, confirming the chronic tendency of this form of CL. Relapses and treatment failures are also not exceptional [23, 24]. Infection caused by *L. tropica* seems to be more insidious compared with *L. major* infection, with a longer incubation period and probably fewer inflammatory lesions [24]. However, several multiple and inflammatory as well as infiltrative diffuse lesions were described in some Moroccan outbreaks [28].

**Figure 4.**
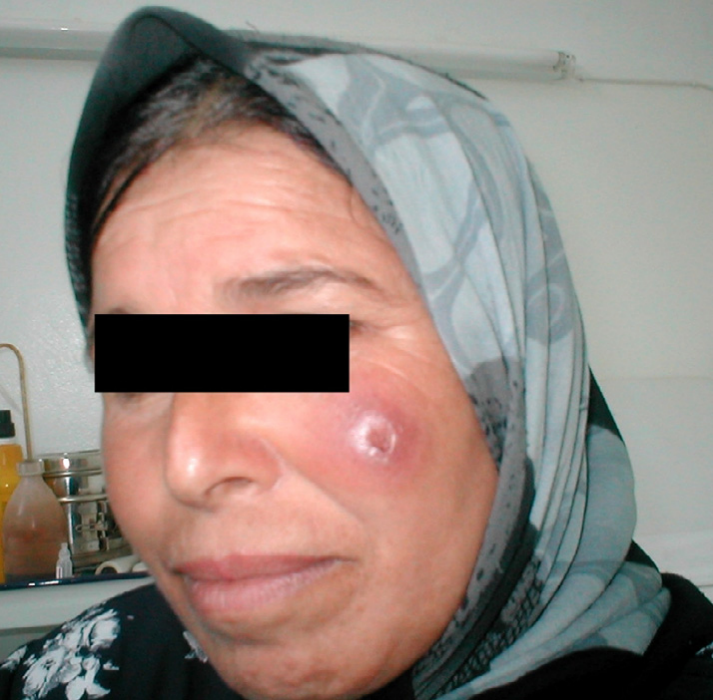
Infiltrated lesion of the face due to *L. infantum*.

Most of the *L. infantum-*infected patients have a single, small, ulcerated, and crusty lesion on the face surrounded by a significant zone of infiltration (Fig. 4) [26].

In NA countries, CL treatment methods mainly depend on clinical features and patients’ access to health care. The pentavalent antimonial (Sb) meglumine antimoniate (Glucantime^®^) is the first-line drug for the three encountered CL forms [7]. According to the CL lesion number and location, Glucantime^®^ is used either as intralesional infiltrations or as intramuscular injections. Intralesional infiltrations are indicated when lesions are less than 5 and/or limb-localized. Two infiltrations per week are usually performed for 2–4 weeks. The recommended dose for intramuscular administration is 20 mg/kg/day for 10–20 days. Alternative drugs are rare in NA countries. The use of fluconazole is limited to the private sector. Cryotherapy has been recently introduced in University Dermatological departments. Liposomal amphotericine B is not yet available. Therapeutic abstention is advised for patients with small healing lesions [7].

## Epidemiological situation and control measures in NA countries

### Tunisia

In Tunisia, CL was historically confined to the oasis of Gafsa and its surroundings (South-West Tunisia) where the disease was typically sporadic and occasionally epidemic, particularly in French soldiers that camped in the Gafsa region in the late 19th century. This cutaneous affection was named “clou de Gafsa”. In 1982, a CL outbreak occurred near the dam of Sidi Saad (Kairouan governorate, Central Tunisia) that had been just finished. A continuous spread to the western, eastern, and southern neighboring areas was then observed during the next few years with emergence of new foci each year. Consequently, a drastic increase in the number of CL cases was observed, with annual case incidence ranging from 1 to 10 thousand cases according to climatic factors and the cyclic immunization of the population. This CL form was identified as ZCL [22]. Currently, ZCL is distributed in Central and Southern Tunisia (Fig. 1), where it occurs as seasonal epidemics and represents the most significant CL form in terms of incidence and morbidity. Although CL is usually self-curing and not life-threatening, individual cases may be psychologically and socially damaging and epidemics are considered as a major public health priority.

Since the onset of epidemics, a national program has been implemented and a guideline for the clinical management of cases has been elaborated. This national program is mainly based on passive case detection, case notification, and free treatment with Glucantime^®^. Control of *P. obesus* and *Meriones* sp. in the neighborhood of villages by manual pulling of chenopods, deep plouwing of colonies of the rodent or by poisoning were also recommended [22]. However, such actions are demanding and expensive, and consequently are often partially and intermittently performed.

Cutaneous leishmaniasis caused by *L. killicki* and CL caused by *L. infantum* are largely less prevalent than ZCL, with an incidence of 50–150 cases per year for each CL form. Cases due to *L. killicki* occur as scarce scattered cases and prevail in communities living in the rocky mountainside of the Tataouine region (South-East) [24] (Fig. 1). However, since 2005, new *L. killicki* foci have emerged in the central and South-Western parts of the country [23] (Fig. 1). Cutaneous leishmaniasis due to *L. infantum* is mainly confined to the Northern part of the country. However, a spread to the center has recently been reported [40] (Fig. 1). *Leishmania infantum* CL cases are usually scattered and rarely clustered, as was reported in the Sidi-Bourouis region in 2001 [8, 48]. No specific control measures are recommended against these two CL sporadic forms.

### Algeria

Cutaneous leishmaniasis has been reported in Algeria since the early 20th century. Its incidence was relatively low and its distribution was mostly restricted to some regions, particularly the oasis of Biskra. Like in Tunisia, a noteworthy increase in ZCL incidence associated with a geographic spread has been observed since the 1980s. Currently, Algeria is considered by the WHO as one of the most affected countries by CL in the world [2].

Cutaneous leishmaniasis caused by *L. major* is the dominant CL form. It is distributed over a wide band across the southern arid zones (Fig. 1). A worrying extension to the North (village of El Mhir) has been recently reported [20]. The most active foci are those of Biskra, M’sila, and Adala. The annual incidence of cases varies from 10,000 to 40,000 cases per year. As in Tunisia, a national control program has been operational since 2005. It is also based on passive case detection and free treatment with Glucantime^®^. The main reservoir of the disease in Algeria being *P. obesus*, a pilot project aiming to remove *Chenopodiaceae* around houses within a radius of 300 m before the transmission season was undertaken in the Msila region in 2003. Over 3,600 ha were treated and the number of cases decreased from 1391 in 2003 to 965 in 2004 [27]. On the other hand, insecticides (Deltamethrin) were used in Algeria on a large scale from 2006 in several governorates, which has led to a significant decline in the incidence rate since 2007 [44].

As reported in Tunisia, CL caused by *L. tropica* and CL caused by *L. infantum* are less prevalent than ZCL. Only a few *L. tropica* CL cases were reported in 2005 in Ghardaia, South-West [42] (Fig. 1). Cases caused by *L. infantum* are sporadic (a few hundred per year) and scattered in the North, especially in Kabylia and Algiers, within classical foci of visceral and canine leishmaniasis [41] (Fig. 1).

### Libya

Most published reports in Libya concern ZCL, which is largely the main form in this country. The main ZCL foci are located in the North-West of the country in Tripoli, Yafran, Djebel Neffoussa, and Nalut regions [30, 31]. A spread to the province of Syrte in the center was recently observed [32] (Fig. 1).

The first case of CL caused by *L. tropica* in Libya was reported in 2006 in a 10-month-old baby in the district of Beni Walid [9]. However, recent molecular identifications revealed that cases due to *L. tropica* are prevalent in many North-Western districts such as Nalut, Misrata, Jabal El Gharbi, and Tarhouna [3, 13] (Fig. 1).

Very few data concerning CL caused by *L. infantum* in Libya are available. This form is probably restricted to the Northern visceral leishmaniasis foci of Tripoli and Djebel El Akhdhar [51].

### Morocco

Since the 1980s, a drastic increase in CL incidence has been observed in Morocco, where the current number of cases varies from 4000 to 6000 per year [57]. In this country, the epidemiological features of CL are quite different from those encountered in other Maghreb countries. In fact, the incidence of *L. tropica* is by far higher in Morocco compared with Tunisia, Algeria, and Libya. The geographical distribution of this species is also larger.

However, ZCL is still the most prevalent form, with occurrence of outbreaks amounting to several thousand cases. The main transmission areas of *L. major* are bordering the Sahara desert of the Eastern parts of the country in a large band including from the North to the South Oujda, Errachidia, Ouarzazate, and Tata [57, 58] (Fig. 1).

*Leishmania tropica* has the largest geographic distribution. Multiple foci are found throughout the center of the country in a band stretching from North to South along the flank of the high and middle Atlas. The main active foci are those of Fes and Taza in the North, Boulemane and Beni Mellal in the center, and Taroudant and Chichaoua in the South [1, 57, 59] (Fig. 1). In these active foci, epidemics of hundreds of cases were reported these last two decades [57]. The emergence of CL due to *L. tropica* as an increasingly important public health problem in Morocco appears to be related to several factors. The spread of the disease might be facilitated by increasing urbanization processes in the settlements, that are usually overcrowded and provide inadequate housing and poor sanitation. Human and dog population movement could act as an important risk factor for the spread of the disease [59].

As in neighboring countries, a Morocco CL program exists. However, the case management approach depends on the causal species. In fact, the coverage of the lesions after Glucantime® treatment is used to limit the transmission of *L. tropica*.

Cutaneous leishmaniasis cases due to *L. infantum* are still rarely identified in Morocco [57]. However, recently molecular CL species characterization identified a new *L. infantum* CL focus (Sidi Kacem) in the North [58] (Fig. 1).

### Egypt

Cutaneous leishmaniasis is not a disease with public health impact in Egypt. The disease incidence remains low despite the high endemicity in all neighboring countries, namely Libya in the West, and Palestine and Jordan in the East. Recent reports reveal that the Sinai Peninsula desert is the main current focus, with sporadic cases of both *L. major* and *L. tropica* [49, 64] (Fig. 1).

## Conclusion

Cutaneous leishmaniasis is one of the main public health problems in Maghreb countries. Socioeconomic and psycho-sanitary impacts are considerable, inducing a productivity gap and an impediment for development. Zoonotic CL is the most prevalent form, with thousands of cases per year. However, ZCL control is difficult regarding the complexity of the *L. major* transmission cycle and the high cost of the few available options. In the absence of a vaccine, control measures should be adapted according to the epidemiological characteristics of the foci concerned. In Morocco, the epidemiology of *L. tropica* CL is distinguishable from that encountered in other NA countries, presenting a higher incidence rate and a putative anthroponotic reservoir. More investigations are needed to understand this model of the transmission cycle better.
